# European Society of Cardiology/European Society of Hypertension versus the American College of Cardiology/American Heart Association guidelines on the cut-off values for early hypertension: a microvascular perspective

**DOI:** 10.1038/s41598-021-83096-1

**Published:** 2021-02-10

**Authors:** H. Shokr, D. Gherghel

**Affiliations:** Vascular Research Laboratory, College of Health and Life Sciences, Aston University, Birmingham, B4 7ET UK

**Keywords:** Cardiology, Diseases, Medical research, Risk factors

## Abstract

The aim of this study was to investigate retinal and peripheral microvascular function in asymptomatic individuals that fall into different BP groups when using either the ESC/ESH or the ACC/AHA guidelines. Retinal and peripheral microvascular function was assessed in 358 participants by means of dynamic retinal vessel analysis and digital thermal monitoring, respectively. Blood pressure and lipid panel were also evaluated. Retinal vascular function measured in all groups belonging to the ACC/ASH classifications were within the normal values for age-matched normal population. Individuals classed as grade 1 hypertension according to the ESC/ESH guidelines, however, exhibited a significantly decreased artery baseline (*p* = 0.0004) and MC (*p* = 0.040), higher slope_AD_ (*p* = 0.0018) and decreased vein MC (*p* = 0.0446) compared to age matched normal individuals. In addition, they also had significant lower artery baseline, artery BDF, MD and MC than individuals classed as stage 1 hypertension based on the ACC/ASH guidelines (*p* = 0.00022, *p* = 0.0179, *p* = 0.0409 and *p* = 0.0329 respectively). Peripheral vascular reactivity (aTR) was lower in ESC /ESH grade I compared to those graded ACC/ASH stage I hypertension (*p* = 0.0122). The conclusion of this study is that microvascular dysfunctions is present at multiple levels only in individuals with ESC/ESH grade 1 hypertension. This observation could be important when deciding personalised care in individuals with early hypertensive changes.

## Introduction

One of the main differences between the latest guidelines published in 2018 by the European Society of Cardiology/European Society of Hypertension (ESC/ESH) and those published in 2017 by The American College of Cardiology/American Heart Association (ACC/AHA) is the cut-off for what is considered elevated blood pressure (BP) and the first stage of hypertension (HTN) diagnosis^1^. As it can be expected, such difference is bound to have a high impact on clinical diagnosis and management of HTN. By using the ACC/AHA guidelines, the number of patients diagnosed with having this disease increased significantly and so did the pressure on the health system and economy. In addition, such change in practice has also a significant impact on the patients’ physical and psychological wellbeing^1^. Nevertheless, it would seem sensible that, in individuals with higher risk for cardiovascular disease (CVD), early diagnosis and interventions are applied at lower BP values. Indeed, both ECC/ESH guidelines and ACC/AHA have recognised this fact and recommended considering treating patients with high CVD risk at a BP threshold lower than their current cut-off for grade/stage 1 HTN^2^. In this way, positive progress is made towards considering individual over population factors when considering someone of being at risk for CVD.

But what is the real measure of a high CVD risk? Today, the gold standard for absolute CVD risk is based on the Framingham 10-year CVD risk score (FRS)^3^. Other risk scores, such as the Prospective Cardiovascular Mὒnster (PROCAM) and the European Society of Cardiology Systematic Coronary Risk Evaluation (SCORE)^4,5^ are also being used for the same purpose. These calculations are based, however, only on few non-modifiable as well as some modifiable risk factors, thus limiting their ability to accurately predict the risk for CVD in all individuals. Indeed, it has been shown that they could either over‐ or underestimate actual risk for CVD in a large number cases^4,6,7^. Consequently, it has been suggested that other factors, such as obesity, psychosocial stress and lifestyle, to name just a few, should also be included for more precise estimations^8^. In addition, vascular endothelial function, a parameter often overlooked, is essential not only for investigations into the pathophysiology of CVD but also for better CVD risk stratification^9–11^. Assessing vascular endothelial function is usually performed using techniques such as ultrasound flow‐mediated dilation (FMD), pulse wave analysis (PWA), plethysmography and iontophoresis^12^. These tests can, however, be complex and time‐consuming, and are performed only in highly specialized services, this contributing to the lack of inclusion of this parameter in the more largely circulated risk scores. Nevertheless, parameters such as retinal microvascular function has been found to show a good association not only with various circulatory markers for CVD^13,14^, but also with other modifiable and non-modifiable risk factors for this disease such as obesity^15^, family history^16^ and age^17,18^. Retinal microvascular function assessments provide instant integrated and dynamic data analysis that is specific to each individual. In addition, it also correlates with peripheral markers for endothelial dysfunction. Indeed, we have recently shown that signs of abnormal vascular function are similarly present and detectable in various microvascular beds, despite existing differences in their anatomical and physiological properties^19^. This observation is important and helps clinician to detect signs of microvascular dysfunction regardless of the method they can access to. By having a choice, practitioners can make better decisions whether to treat or not selected patients with lower BP values but with additional CVD risk, thus making decisions based on individual, rather that population risk factors, an important step towards personalised management of HTN. Nevertheless, in individuals without higher risk, the decision to treat might not be always justified. In order to shed a possible light on the validity of lowering the cut-off for the diagnosis of early hypertension in individuals without additional CVD risk, this study aimed to analyse the retinal and peripheral microvascular function in subjects that fall into different BP groups when using either the ESC/ESH or the ACC/AHA guidelines^2,20^.

## Methods

### Study participants

Healthy individuals over the age of 30 were recruited through advertisements at the Vascular Research Laboratory, Aston University (Birmingham, UK). Ethical approval was sought from the relevant local ethics committee (Aston University's Ethics Committee), and written informed consent was received from all participants prior to study enrolment. The study was designed and conducted in accordance with the tenets of the Declaration of Helsinki, and all study‐related procedures adhered to institutional guidelines^14,17^.

Study exclusion criteria were defined as the positive diagnosis of hypertension, CVD, cerebrovascular disease, peripheral vascular disease, dyslipidaemia, diabetes, as well as other metabolic disorders or chronic diseases that required treatment. Individuals using any vasoactive medications were also excluded from the study. Potential participants were also screened for ocular diseases and were excluded from the study if they had a refractive error of more than ± 3DS and more than ± 1DC equivalent, intra‐ocular pressure (IOP) greater than 21 mmHg, cataract or any other media opacities, as well as history of intra‐ocular surgery or any form of retinal or neuro‐ophthalmic disease affecting the ocular vascular system^14,18^.

### General investigations

Participants who met the inclusion criteria were invited to a 2-h visit. Initially, they were requested to complete a general health history questionnaire, also detailing daily diet, physical activity and alcohol consumption. All study‐related measurements were performed between 8 and 11 am following a 12‐hr overnight fast, which included refraining from alcohol and caffeine.

Standard anthropometric measures of height and weight were recorded to determine body mass index (BMI = weight/height^2^)^14^.

### Blood pressure assessment and patients grouping

Measurements of BP were performed on two separate occasions, one in-clinic and one out-of-clinic^2,20^. During the in-clinic visit, BP was measured in a quiet environment, with the patient seated for 5 min, 3 times at 1–2 min intervals using an automatic BP monitor (UA‐767; A & D Instruments Ltd, UK) and an adequate cuff size. The BP values were confirmed by a second measurement using a computer‐operated ambulatory BP and electrocardiogram (ECG) monitor (Cardiotens‐01, Meditech Ltd, Budapest, Hungary). All subjects maintained their normal activity and were carefully instructed to complete a diary each time their activities changed, or when any chronic medication was taken. The 24‐h BP were later downloaded and analysed using the ‘Medibase’ software program (Meditech). SBP as well as diastolic DBP measurements were calculated for the day-time (6 am to 10 pm) and night-time (10 pm to 6 am) intervals. At least 80% of the programmed recordings were required for a diurnal curve to be considered in the present analysis^14,19^.

Using the day-time SBP and DBP values, study participants were stratified into three subcategories: “normal”, “high normal” and ‘’Grade I’’ as recommended by the 2018 ESC/ESH Guidelines. They were then also further classified into three other subcategories: “normal”, “elevated” and “stage I” as recommended by the 2017 ACC/AHA guidelines. Subjects classed as “optimal” according to the 2018 ESC/ESH guidelines were excluded and their values were included in our pool of “normal” data^14^.

### Dynamic retinal vessel analysis

**Figure 1:**
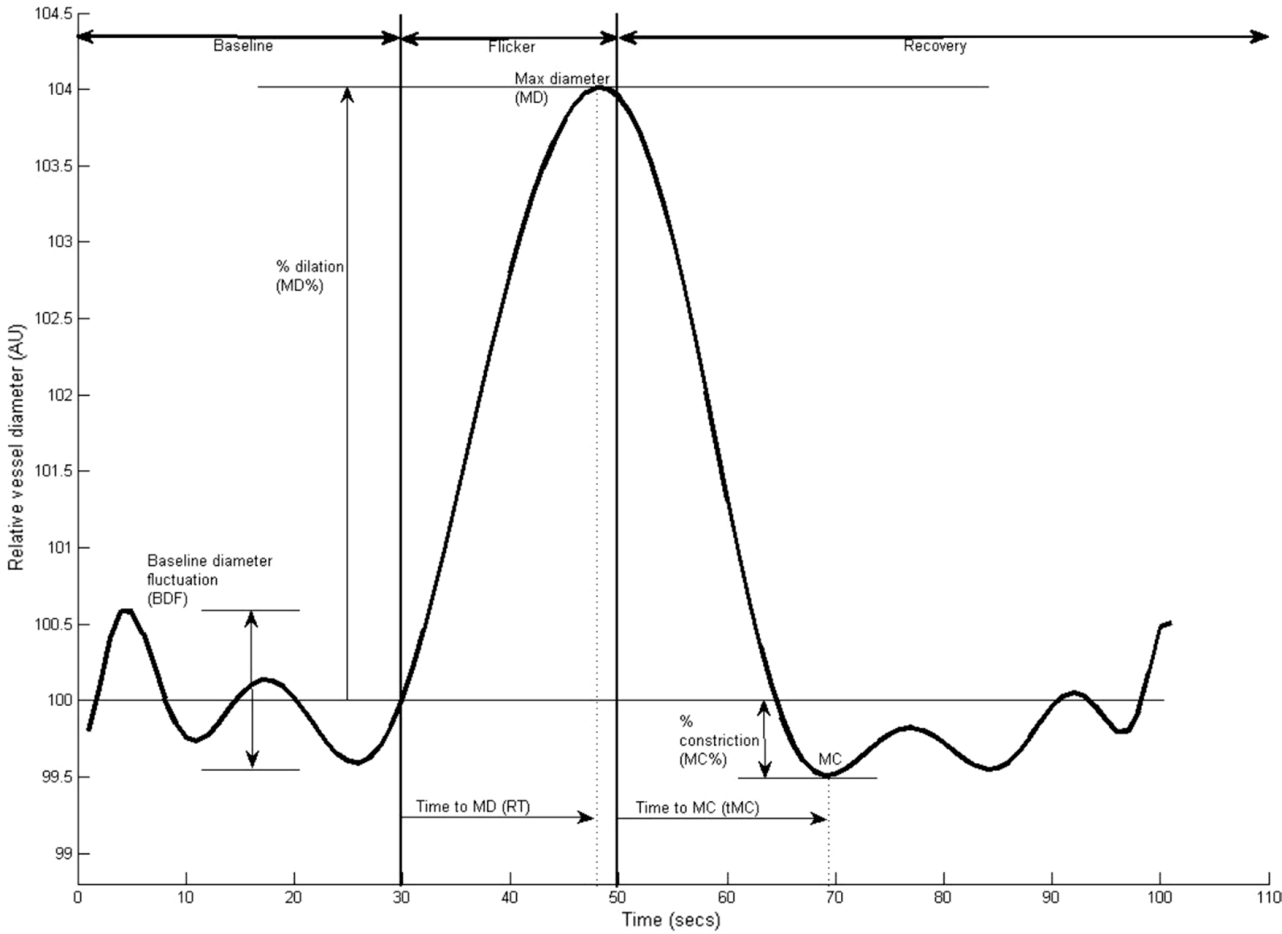
Graphical presentation of the dynamic vessel response profile displaying the parameters calculated and used in analysis. (DA) calculated as (MD-MC). (MD%) calculated as the percent increase from baseline to MD. (MC%) calculated as the percent constriction below baseline following MD.

Retinal vessel reactivity was assessed using the dynamic retinal vessel analyser (DVA, IMEDOS GmbH, Jena, Germany) in accordance with an established protocol^21^. All measurements were performed in a temperature-controlled environment (22 °C) following pupil dilation with 1% Tropicamide (Chauvin Pharmaceuticals Ltd, UK) and were taken from the inferior temporal vessel branches approximately one and a half disc diameters from the optic nerve head of one unselected eye. Using a validated in-house algorithm, the following vessel reactivity and time-course parameters were determined for each flicker cycle and then averaged over the 3 cycles, with the artery and vein regarded separately as follows: the average baseline diameter and range of maximum and minimum baseline vessel diameters (baseline diameter fluctuation, BDF); the maximum vessel dilation diameter during flicker stimulation expressed as a percentage change relative to baseline diameter (MD%) and the time taken in seconds to reach the maximum diameter (tMD); the maximum vessel constriction diameter during the postflicker recovery period expressed as a percentage change relative to baseline diameter (MC%) and the time taken in seconds to reach the maximum vessel constriction diameter (tMC); the overall dilation amplitude (DA) calculated as the difference between MD and MC; and the baseline-corrected flicker response (BCFR) used to describe the overall dilation amplitude after normalizing for fluctuations in baseline diameters (DA-BDF). In addition, the arterial (A) and venous (V) dilation slopes (Slope_AD/VD_ = (MD − baseline diameter) / tMD) and constriction slopes (Slope_AC/VC_ = (MC − MD) / tMC) were also calculated (Fig. 1)^16^. The sensitivity and reproducibility of DVA assessments in healthy subjects has been reported previously and the normal expected retinal responses to flicker-light stimulation have been reported to be around 6.9 ± 2.8% in arteries and 6.5 ± 2.8% in veins^22,23^.

### Digital thermal monitoring (DTM)

**Figure 2:**
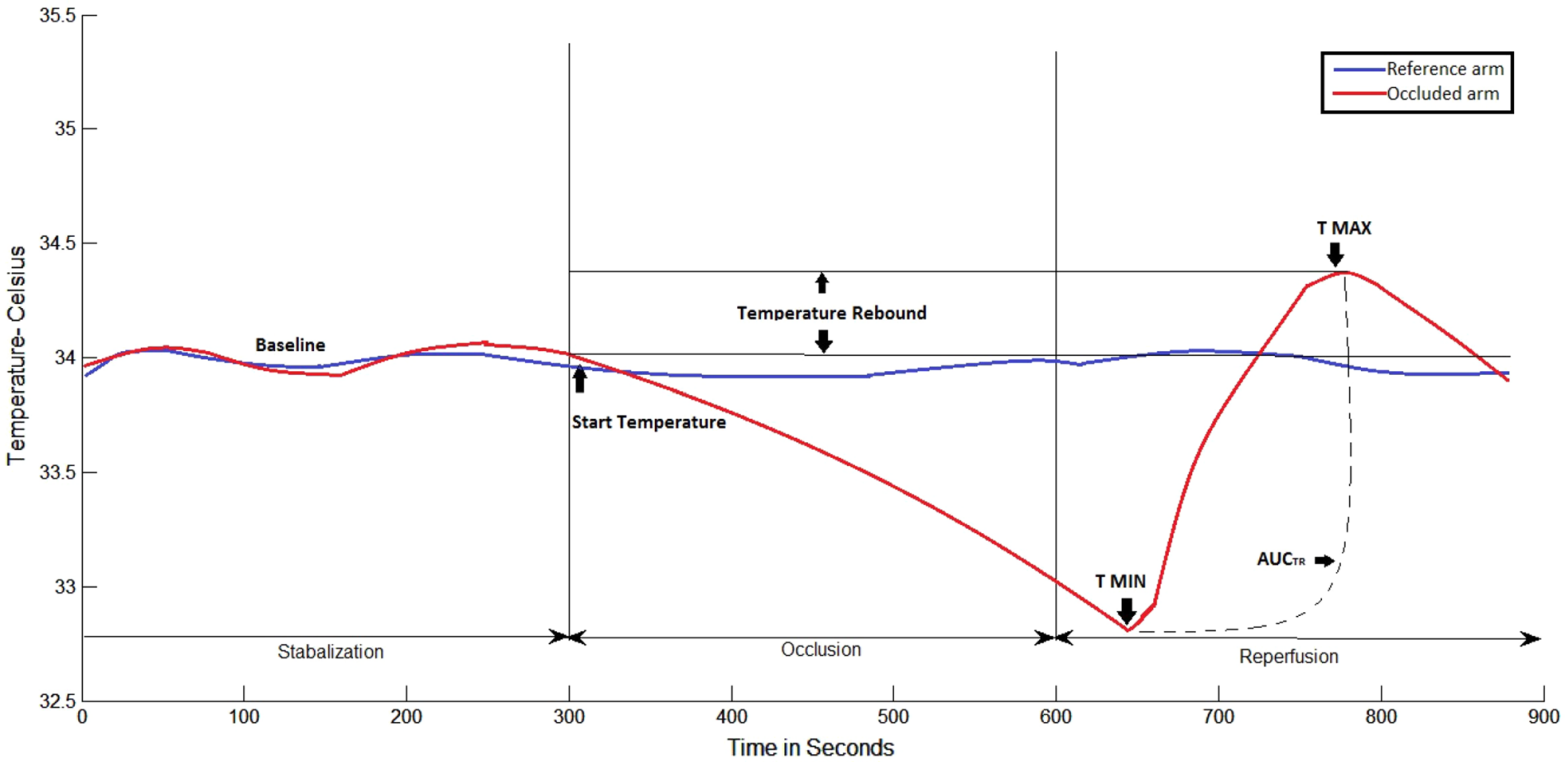
Graphical representation of the Digital Thermal Monitor software analysis. T MAX, maximum temperature; TMIN, minimum temperature; AUCTR, Area under the curve temperature.

The peripheral microvascular reactivity at the level of the fingertips was assessed using VENDYS 5000 BCE DTM system (Endothelix, Inc, Houston, TX, USA) according to an established protocol^24^. Figure 2 shows a representative example of a temperature–time trace and the primary DTM-derived measures, related to thermal debt and recovery that were recorded and calculated.

Temperature rebound (TR): maximum temperature − start temperature (just before cuff inflation); adjusted temperature rebound (aTR): temperature rebound/start temperature; area under the curve temperature rebound (AUCTR): area under the curve between maximum temperature and minimum temperature. The post-occlusive adjusted temperature rebound aTR determined by the software algorithm is directly associated with the extent of the subjects vascular reactivity^25^. An aTR below 1 is considered poor cardiovascular reactivity, whereas an aTR between 1 and 2 is considered intermediate vascular reactivity and an aTR of > 2 is considered healthy vascular reactivity.

### Statistical analysis

All data are reported as mean (SD) unless otherwise indicated. The Shapiro–Wilk test was used to determine the distribution of the data. Univariate associations were determined using Pearson's (normally distributed data) or Spearman's method (non‐normally distributed data), and forward stepwise regression analyses were performed to test the influences of systemic and circulating markers on the measured variables. Differences between groups were subsequently assessed using one‐way analysis of variance (anova) or analysis of covariance (ancova). *p* values of < 0.05 were considered significant. All analyses were performed using Statistica software.

## Results

A total number of 372 participants were initially screened for study inclusion of which 14 individuals were excluded based on the quality of retinal vascular image analysis or to the fact that they did not tolerate the ambulatory BP measurements. The remaining 358 participants were included in the final analysis and classified into 3 study groups using the current ESC/ESH Guidelines (normal BP:66 individuals,; high normal BP: 40 individuals; and grade 1 hypertension: 53 individuals). A further three groups were decided based on the ACC/AHA guidelines: (normal BP: 83 individuals; Elevated BP: 46 individuals; and stage 1 hypertension: 70 individuals).

General characteristics of the study population are presented in Table 1. There were no significant differences in age, gender, BMI, HR, IOP, glucose, TG, cholesterol, LDL-c, HDL-c and body composition between all the study groups (all *p* > 0.05). There was a significant difference between groups in SBP and DBP (*p* < 0.001). In addition, there was also significant differences between the peripheral vascular reactivity (aTR) between ESC /ESH grade I compared to ACC/ASH stage I hypertension, with individuals graded as ESC /ESH grade I having lower aTR than those graded ACC/ASH stage I hypertension (*p* = 0.0122).

**Table 1:** General characteristics of the study population.

	ACC/AHA Normal < 120& < 80	ESC/ESH Normal SBP:120–129 DBP:80–84	*p *value	ACC/AHA Elevated SBP: 120–129 DBP: < 80	ESC/ESH High Normal SBP:130–139 DBP:85–89	*p* value	ACC/AHA Stage I SBP:130–139 DBP:80–89	ESC/ESH Grade I SBP:140–159 DBP:90–99	*p *value
Number	83	66	–	46	40	–	70	53	–
Gender	30 M:53F	34 M:32F	–	29 M:17F	22:18F	–	44 M:37F	32 M:21F	–
Age (years)	40.1.8 (1.35)	43.91 (1.70)	0.0859	42.83 (2.31)	45.80 (2.53)	0.3897	44.88 (3.4)	48.77 (2.7)	0.1063
SBP(mmHg)	109.43 7.08)	122.52 (4.98)	0.0000*	124.17 (6.88)	131.03 (6.74)	0.0002*	134.61 (8.72)	142.00 (7.52)	0.0000*
DBP(mmHg)	68.33 (6.15)	76.15 (5.92)	0.0000*	72.76 (4.98)	83.93 (5.37)	0.0000*	83.95 (4.71)	89.47 (6.54)	0.0000*
HR (bpm)	68.055 (9.28)	65.06 (10.15)	0.0659	63.31 (9.38)	67.37 (10.85)	0.1085	68.37 (10.87)	69.59 (10.93)	0.5467
IOP	13.30 (2.34)	13.41 (2.64)	0.8139	13.25 (2.59)	13.43 (2.25)	0.7123	14.33 (2.63)	15.29 (3.01)	0.0970
BMI (kg/m^2^)	24.69 (0.36)	25.66 (0.45)	0.0934	26.73 (0.71)	25.70 (0.78)	0.3175	26.49 (4.20)	28.09 (4.40)	0.0680
Body fat %	28.00 (1.68)	23.76 (1.76)	0.0864	23.02 (1.90)	28.31 (2.06)	0.0670	27.65 (2.43)	31.93 (3.97)	0.3651
Body water %	52.51 (1.65)	51.56 (1.73)	0.6907	54.25 (2.30)	48.84 (2.48)	0.1180	46.80 (2.96)	49.44 (4.84)	0.6445
Muscle mass %	47.45 (2.59)	50.89 (2.72)	0.1673	60.94 (3.13)	55.68 (3.38)	0.2599	52.75 (4.29)	43.30 (6.10)	0.2583
Glucose (mmol/L)	4.87 (0.55)	4.93 (0.86)	0.6178	5.07 (0.82)	5.03 (0.60)	0.8433	4.93 (0.92)	5.15 (1.31)	0.3356
TG (mmol/L)	1.139 (0.56)	1.19 (0.61)	0.6345	1.15 (0.48)	1.25 (0.46)	0.4397	1.263 (0.62)	1.44 (0.80)	0.2210
T.Chol (mmol/L)	4.40 (0.78)	4.70 (1.02)	0.5642	4.88 (1.07)	4.55 (0.91)	0.2040	4.50 (0.89)	4.72 (0.86)	0.2516
HDL-C (mmol/L)	1.33 (0.41)	1.37 (0.39)	0.5212	1.35 (0.38)	1.23 (0.58)	0.3288	1.33 (0.56)	1.21 (0.44)	0.2848
LDL-C (mmol/L)	2.57 (0.91)	2.78 (0.12)	0.1639	2.10 (1.09)	2.82 (1.05)	0.5199	2.59 (0.94)	2.90 (0.93)	0.1131
TG/HDL	1.00 (0.81)	1.01 (0.81)	0.9561	0.98 (0.63)	1.35 (0.91)	0.0683	1.21 (0.92)	1.36 (0.94)	0.4460
aTR	1.60 (0.13)	1.93 (0.11)	0.0365*	1.70 (0.11)	1.63 (0.11)	0.7790	1.80 (0.12)	1.20 (0.20)	0.0122*

Abbreviations: SBP: systolic blood pressure; DBP: diastolic blood pressure; HR: heart rate; IOP: intraocular pressure; BMI: body mass index; TG: triglycerides; T. Chol: total cholesterol; HDL-C: high-density lipoprotein cholesterol; LDL-C: low-density lipoprotein; aTR: adjusted temperature rebound *Significant *p* values are indicated where *p* < 0.05 was considered significant.

Comparisons of group differences in measured retinal arterial and venous DVA parameters after flicker stimulation are presented in Tables 2 and 3. All values reported are based on data averaged across the three flicker cycles with arteries and veins considered separately. Values were further compared to the average normal values for the groups’ corresponding age range.

**Table 2:** Summary of retinal arterial vascular function parameters.

Normal Average	ACC/AHA Normal < 120& < 80	ESC/ESH Normal SBP:120–129 DBP:80–84	ANOVA/ANCOA *p* value	ACC/AHA Elevated SBP:120–129 DBP: < 80	ESC/ESH High Normal SBP:130–139 DBP:85–89	ANOVA/ANOVA *p* value	ACC/AHA Stage I SBP:130–139 DBP:80–89	ESC/ESH Grade I SBP:140–159 DBP:90–99	ANOVA/ANCOVA *p* value
Artery baseline 99.99 (0.002)	99.98 (0.12)	99.88 (0.77)	0.1979	99.83 (0.95)	99.97 (0.92)	0.4346	99.99 (0.62)	97.93 (0.27)	0.0002*
Artery-BDF 5.66 (2.63)	5.64 (2.650)	6.14 (3.46)	0.3461	6.28 (2.87)	5.83 (2.74)	0.5250	5.98 (3.42)	5.02 (2.41)	0.0179*
Artery-MD 103.67 (2.20)	103.57 (2.20)	104.07 (2.24)	0.2000	104.44 (2.2)	104.19 (2.74)	0.5780	104.14 (2.43)	102.75 (4.26)	0.0409*
Artery-tMD 22.14 (10.09)	21.88 (9.63)	20.87 (6.27)	0.5047	20.84 (5.83)	20.27 (6.44)	0.7120	19.86 (6.74)	20.10 (7.11)	0.9493
Artery-MDp 3.67 (2.21)	3.59 (2.20)	4.20 (2.53)	0.1400	4.64 (2.63)	4.48 (2.75)	0.7901	4.15 (2.44)	4.01 (2.55)	0.1020
Artery-MC 97.51 (1.89)	97.73 (0.19)	97.64 (0.25)	0.7691	97.83 (1.82)	97.53 (2.15)	0.5410	97.58 (1.80)	96.08 (4.97)	0.0329*
Artery-tMC 27.05 (8.31)	27.47 (7.36)	27.88 (6.98)	0.7455	28.09 (6.60)	28.05 (6.44)	0.9810	27.49 (6.70)	28.85 (7.96)	0.3641
Artery-MCp − 2.49 (1.89)	− 2.22 (1.84)	− 2.056 (1.87)	0.6124	− 1.99 (1.95)	− 2.14 (2.14)	0.3810	− 2.41 (1.80)	− 1.94 (1.72)	0.2002
Artery-DA 6.16 (2.95)	5.77 (2.87)	6.17 (2.96)	0.5191	6.61 (2.95)	7.30 (3.63)	0.4310	6.57 (3.02)	6.71 (2.91)	0.8096
Artery-BCFR 0.56 (2.73)	0.30 (2.57)	0.26 (2.97)	0.9244	0.43 (2.74)	0.44 (3.02)	0.1681	0.67 (3.07)	0.89 (3.12)	0.7326
Artery-Slope_AD_ 0.21 (0.13)	0.21 (0.16)	0.25 (0.15)	0.1347	0.27 (0.16)	0.26 (0.32)	0.1902	0.28 (0.20)	0.35 (0.30)	0.2335
Artery-Slope_AC_ − 0.36 (0.30)	− 0.36 (0.45)	− 0.30 (0.22)	0.3600	− 0.31 (0.22)	− 0.32 (0.18)	0.9603	− 0.29 (0.20)	− 0.27 (0.51)	0.2826

Abbreviations: ANOVA, analysis of variance; ANCOVA, analysis of covariance; Baseline, baseline diameter; BDF, baseline diameter fluctuation; BCFR, Baseline corrected flicker response; tMD, time to reach MD; MDp, percent dilation; tMC, time to reach MC; MC (%), percent constriction; DA, dilation amplitude (difference between MD and MC during flicker) Slope_AD_, slope of arterial dilation; Slope_AC_, slope of arterial constriction. *Significant *p* values are indicated where *p* < 0.05 was considered significant.

**Table 3:** Summary of retinal arterial vascular function parameters.

Normal Average	ACC Normal < 120& < 80	ESC Normal SBP:120–129 DBP:80–84	ANOVA/ANCOVA *p* value	ACC Elevated SBP:120–129 DBP: < 80	ESC High Normal SBP:130–139 DBP:85–89	ANOVA/ANCOVA *p* value	ACC Stage I SBP:130–139 DBP:80–89	ESC Grade I SBP:140–159 DBP:90–99	ANOVA/ANCOVA *p* value
Vein-Baseline 99.98 (0.15)	99.96 (0.20)	99.90 (0.78)	0.3996	99.83 (0.97)	99.99 (0.002)	0.3733	99.99 (0.0025)	99.91 (0.0022)	0.4251
Vein-BDF 4.39 (2.04)	4.427 (2.22)	3.94 (2.02)	0.2059	4.09 (2.22)	5.149 (3.48)	0.1583	4.70 (2.74)	4.92 (2.48)	0.7667
Vein-MD 104.34 (2.26)	104.19 (2.16)	104.06 (2.24)	0.7239	104.01 (2.19)	105.31 (2.83)	0.5790	105.10 (2.73)	104.61 (2.245)	0.6224
Vein-tMD 22.55 (6.93)	22.37 (6.90)	21.53 (7.40)	0.5058	22.68 (8.62)	21.25 (6.46)	0.5343	19.82 (5.45)	20.43 (5.81)	0.4213
Vein-MDp 4.36 (2.26)	4.24 (2.15)	4.19 (2.61)	0.9114	4.21 (2.75)	5.310 (2.93)	0.1326	5.10 (2.73)	4.612 (2.24)	0.5127
Vein MC 98.71 (1.27)	98.87 (1.14)	99.20 (1.12)	0.0957	98.36 (1.02)	98.70 (1.51)	0.2408	98.46 (1.123)	97.13 (12.5)	0.0383*
Vein-tMC 31.68 (5.51)	31.23 (5.62)	30.15 (6.38)	0.2990	31.26 (6.61)	30.54 (6.34)	0.6757	30.37 (5.76)	31.0384 (5.20)	0.6642
Vein-MCp − 1.27 (1.28)	− 1.09 (1.18)	− 0.70 (1.55)	0.0744	− 0.85 (0.70)	− 1.29 (0.81)	0.1973	− 1.17 (1.25)	− 1.06925902	0.5289
Vein-DA 5.63 (2.88)	5.325 (2.69)	4.85 (2.81)	0.3302	4.86 (1.69)	6.60 (3.20)	0.0869	6.27 (3.16)	5.6811 (2.60)	0.4637
Vein-BCFR 1.23 (2.19)	0.94 (2.37)	0.98 (2.86)	0.9383	0.66 (2.79)	1.56 (2.84)	0.2202	1.52 (2.49)	0.76220 (1.97)	0.2591
Vein-Slope_AD_ 0.23 (0.13)	0.23 (0.14)	0.24 (0.16)	0.5595	0.24 (0.175)	0.50 (1.109)	0.1881	0.39 (0.74)	0.2697 (0.13)	0.4007
Vein-Slope_VC_ − 0.24 (0.15)	− 0.23 (0.14)	− 0.20 (0.13)	0.2551	− 0.20 (0.13)	− 0.27 (0.16)	0.1062	− 0.25 (0.17)	− 0.2318 (0.16)	0.7306

Abbreviations ANOVA, analysis of variance; ANCOVA, analysis of covariance; Baseline, baseline diameter; BDF, baseline diameter fluctuation; BCFR, Baseline corrected flicker response; tMD, time to reach MD; MD (%), percent dilation; tMC, time to reach MC; MC (%), percent constriction; DA, dilation amplitude (difference between MD and MC during flicker) Slope_AD_, slope of venous dilation; Slope_VC_, slope of venous constriction. *Significant *p* values are indicated where *p* < 0.05 was considered significant.

### Retinal vascular response

**Figure 3:**
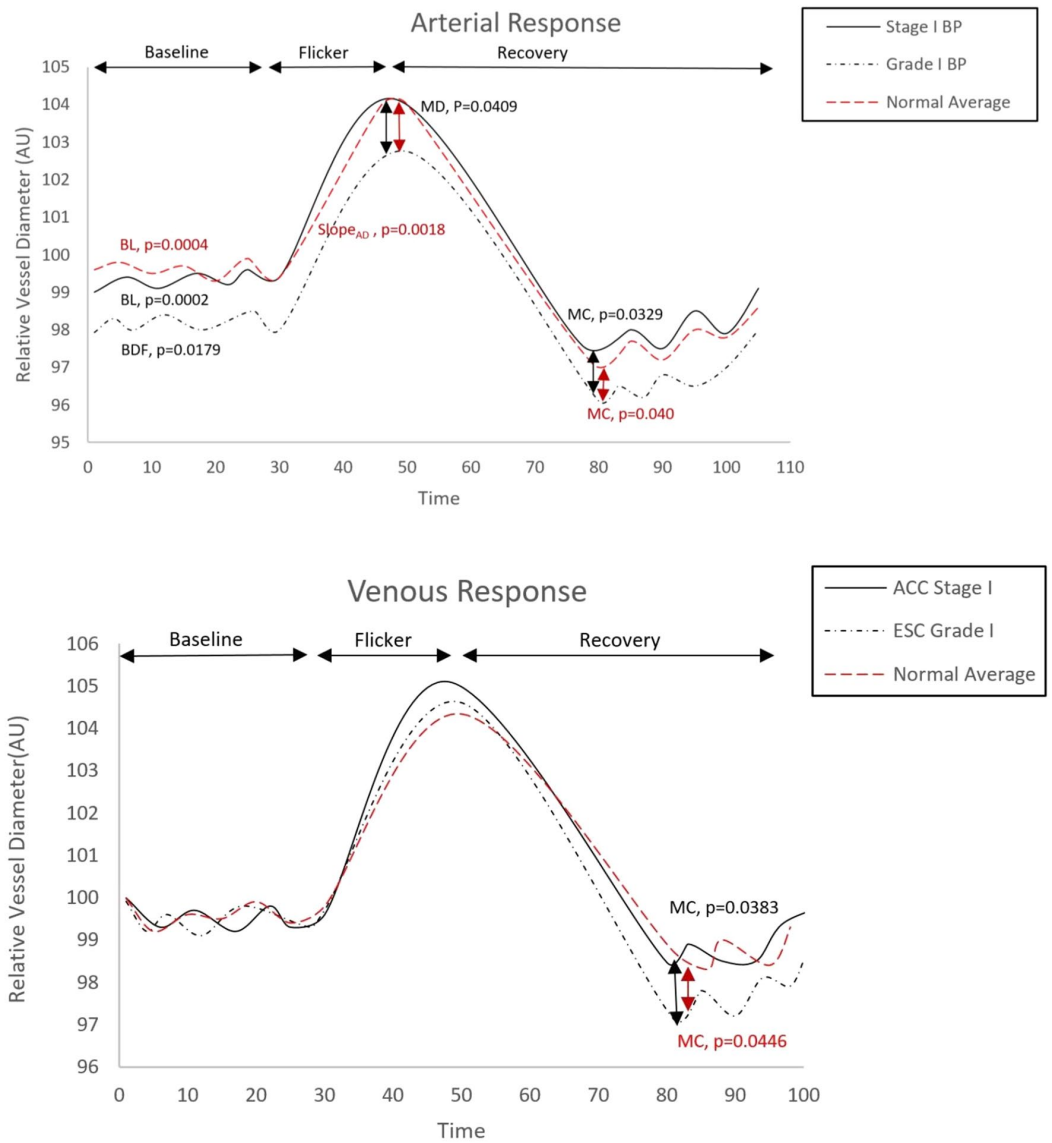
Comparison of retinal arterial and venous response profile across groups. AU, arbitrary units; BDF, baseline diameter fluctuation calculated as the maximum range in vessel diameter during first 30 s of baseline readings; MD%, calculated as the percentage change in vessel diameter from baseline to maximum following onset of flicker; tMD, time to reach maximum diameter during flicker; tMC, time to reach maximum constriction post flicker; slope_AC_ & Slope_VC_, calculated as (MC-MD)/(tMC).

After controlling all the influential covariates identified in multivariate analysis, there were no statistically significant differences in baseline diameter, BDF, BCFR, tMD and tMC, slope_AD_ and slope_AC_ between the ACC/AHA and ESC/ESH normal BP groups (*p* > 0.05, Table 2). Similarly, there were no significant group differences found between ACC/AHA elevated and ESC/ESH high normal BP groups (*p* > 0.05, Table 2). However, individuals classed as grade 1 hypertension according to the ESC/ESH guidelines had significant lower artery baseline, artery BDF, MD and MC than individuals classed as stage 1 hypertension based on the ACC/ASH guidelines (*p* = 0.0002, *p* = 0.0179, *p* = 0.0409 and *p* = 0.0329 respectively). In addition, while the retinal vascular reactivity parameters measured in individuals classed as stage 1 hypertension according to ACC/ASH guidelines were still within the normal limits for their age, the ESC/ESH grade I hypertensive group exhibited a significantly decreased artery baseline (*p* = 0.0004) and MC (*p* = 0.040) as well as higher slope_AD_ (*p* = 0.0018) compared to age matched population average (Table 2; Fig. 3). Similarly, vein MC was significantly decreased in ESC /ESH grade I compared to ACC/ASH stage I hypertension (*p* = 0.0383). In addition, vein MC was significantly decreased only in the ESC/ESH grade I hypertension compared to age matched population average (*p* = 0.0446) (Table 3; Fig. 3).

No significant group differences in any of the other measured arterial DVA parameters were identified (all *p* > 0.05).

## Discussion

The present study looked at the peripheral and retinal microvascular function in various BP groups divided according to either the ESC/ESH or the ACC/AHA guidelines. Our report shows, for the first time, that independent of all systemic influences, retinal microvascular dysfunction firstly manifest itself only in individuals classed as ESC/ESH grade 1 hypertension. In contrast, subjects classed as ACC/AHA stage 1 hypertension exhibited normal retinal microvascular behaviour after provocation using flickering light. In addition, ESC/ESH grade 1 hypertension individuals also exhibited lower peripheral vascular reactivity than those graded as ACC/AHA stage 1 hypertension.

It is well known that microvascular dysfunction is a parameter highly predictive of cardiovascular events^26^. Due to this quality, its early identification is extremely important, especially in individuals at risk for CVD, including those with borderline BP values or classed as grade/stage 1 hypertension. Indeed, similar to what is known for so long in the literature about the effects of systemic hypertension on microvasculature^27^ in the present study we have shown that individuals classed as ESC/ESH grade 1 hypertension demonstrated abnormal peripheral and retinal microvascular function. Indeed, at the retinal vascular level, both vasodilation and vasoconstriction of the retinal arteries and veins were affected. Nevertheless, as all patients with essential hypertension are known to demonstrate microvascular endothelial dysfunction from early stages^28^, it is puzzling how individuals classed as ACC/AHA stage 1 hypertension failed to show such abnormalities. This is an important observation that requires further careful consideration since changes in diagnostic cut-off for systemic hypertension towards lower BP values in individuals without additional risk factors has a very high impact not only on the clinical practice and economy but also on each individual well-being^1^.

Beside its direct effect on the vascular functionality, hypertension is also known to cause an acceleration of the ageing process of the vascular function^29^. We have previously demonstrated that, in ageing, normal individuals there is a decline in retinal vasoregulative capacity in both dilatory and constrictory phases that was independent of any systemic influences^17^ and we have linked this abnormal response to the possibility of an age-related increase in oxidative stress^19^. Nevertheless, the subjects included in the current paper were much younger that those included in our ageing studies. It is possible that, the abnormal microvascular function observed in individuals classed as ESC/ESH grade 1 hypertension was indeed due to a reduced nitric oxide (NO) availability through excess inhibition by reactive oxygen species (ROS)^30^; however, in this case, the main culprit for ROS excess was not ageing but, possibly, an increased BP. Indeed, it has been shown that, in hypertensive patients, NO availability is reduced early in the course of the disease^29^. Although we did not measure either the level of NO or antioxidative markers in our current cohort of patients, in the light of our previous discovery showing that retinal microvascular dysfunction occurs in parallel with changes observed in the level of oxidative stress both in normal^19^ and individuals with abnormal BP levels^15^, it is logic to presume that similar abnormalities occurred in our current cohort of individuals with ESC/ESH grade 1 hypertension, thus contributing to the abnormal retinal microvascular function measured in these individuals.

Our ESC/ESH grade 1 hypertension also exhibited abnormal retinal venous response to flicker. In previous studies, we have already documented that individuals with other categories of cardiovascular risk also exhibit abnormal retinal venous responses to flicker-induced provocation^14,16,31,32^. Structural retinal venular dilation has previously implicated as a strong prediction of adverse CVD events^33,34^. Nevertheless, the abnormal functional retinal venous responses, as measured by DVA, are still to be confirmed as signs of endothelial dysfunction in their own right or as epiphenomena of retinal arterial dysfunction^14,17^. In this particular context, our observed retinal venular dysfunctionality could reflect a compensatory adaptation following sustained arterial dilation during flicker. Further investigation is required to understand the relevance of this kind of response; however, it could be hypothesized that a change in venous calibre associated with either structural or endothelial irregularities could also be used as a marker for cardiovascular risk in individuals with early hypertension^14,16,17^.

In addition to a general retinal microvascular dysfunction, our cohort of individuals with ESC/ESH grade 1 hypertension also exhibited abnormal peripheral microvascular reactivity as assessed by DTM, showing that abnormal microvascular function is a general process in these individuals and can be detected al multiple levels despite differences in vascular physiology and methodologies.

This study found microvascular dysfunctions to be present at multiple levels only in individuals with ESC/ESH grade 1 hypertension and not in those classed as stage 1 hypertension according to the latest ACC/AHA guidelines. The implications of these observations could be important, especially when clinicians decide their intervention based only on controversial, borderline BP values measured in individuals without other CVD risk.

## Data Availability

The data that support the findings of this study are available on request from the corresponding author.
